# Methylation biomarkers for pleomorphic lobular breast cancer - a short report

**DOI:** 10.1007/s13402-015-0241-9

**Published:** 2015-09-21

**Authors:** Cathy B. Moelans, Eva J. Vlug, Cigdem Ercan, Peter Bult, Horst Buerger, Gabor Cserni, Paul J. van Diest, Patrick W. B. Derksen

**Affiliations:** 1 Department of Pathology, University Medical Center Utrecht, PO Box 85500, 3508 GA Utrecht, The Netherlands; 2 Department of Pathology, Radboud University Nijmegen, Nijmegen, The Netherlands; 3 Institute of Pathology, Paderborn, Germany; 4 Department of Pathology, Bács-Kiskun County Teaching Hospital, Kecskemét, Hungary

**Keywords:** Sporadic breast cancer, Lobular breast cancer, Pleomorphic lobular breast cancer, DNA hypermethylation, MS-MLPA, Epigenetics

## Abstract

**Background:**

Pleomorphic invasive lobular cancer (pleomorphic ILC) is a rare variant of ILC that is characterized by a classic ILC-like growth pattern combined with an infiltrative ductal cancer (IDC)-like high nuclear atypicality. There is an ongoing discussion whether pleomorphic ILC is a dedifferentiated form of ILC or in origin an IDC with a secondary loss of cohesion. Since gene promoter hypermethylation is an early event in breast carcinogenesis and thus may provide information on tumor progression, we set out to compare the methylation patterns of pleomorphic ILC, classic ILC and IDC. In addition, we aimed at analyzing the methylation status of pleomorphic ILC.

**Methods:**

We performed promoter methylation profiling of 24 established and putative tumor suppressor genes by methylation-specific multiplex ligation-dependent probe amplification (MS-MLPA) analysis in 20 classical ILC, 16 pleomorphic ILC and 20 IDC cases.

**Results:**

We found that pleomorphic ILC showed relatively low *TP73* and *MLH1* methylation levels and relatively high *RASSF1A* methylation levels compared to classic ILC. Compared to IDC, pleomorphic ILC showed relatively low *MLH1* and *BRCA1* methylation levels. Hierarchical cluster analysis revealed a similar methylation pattern for pleomorphic ILC and IDC, while the methylation pattern of classic ILC was different.

**Conclusion:**

This is the first report to identify *TP73*, *RASSF1A*, *MLH1* and *BRCA1* as possible biomarkers to distinguish pleomorphic ILC from classic ILC and IDC.

## Introduction

Invasive lobular breast cancer (ILC) is the second most prevalent histological breast cancer type that accounts for 10–15 % of all breast cancers [1, 2]. ILC differs from invasive ductal carcinoma (IDC) in biology, histology, clinical presentation and response to therapy (reviewed in [3]). In contrast to ductal tumors, most lobular tumors show loss of E-cadherin expression, which often results from inactivating gene mutation and subsequent loss of heterozygosity or promoter hypermethylation [4]. Indeed, conditional knock-out mouse models have shown that somatic inactivation of E-cadherin leads to ILC development and progression [5, 6]. Among the eight different ILC variants described, classic ILC and pleomorphic ILC are the most common ones [3, 7]. Although the exact frequency of these ILC subtypes has not extensively been documented, approximately 60 % of all ILC cases is classic and approximately 13 % is pleomorphic (reviewed in [3]). Phenotypically, classic ILC is composed of small regular low grade and dissociated cells with intra-cytoplasmic vacuoles and small nuclei that exhibit a highly trabecular infiltrative growth pattern, often distributed in targetoid patterns around uninvolved ducts [8]. Pleomorphic ILC shows a similar growth and invasion pattern, but is composed of high grade polygonal cells with eccentric and highly pleomorphic nuclei. Furthermore, pleomorphic ILC tumors have been reported to be significantly larger than classic ILC tumors, and pleomorphic ILC patients often present with lymph node involvement and a higher rate of metastatic disease compared to classical ILC [9]. Moreover, the overall survival and recurrence rates of pleomorphic ILC patients are worse compared to classic ILC patients [10], indicating that pleomorphic ILC is a more aggressive form of breast cancer than classic ILC.

At the molecular level, classic and pleomorphic ILCs show similarities and differences. Both variants lack expression of basal markers like cytokeratin (CK)5 and CK14, but expresses the luminal epithelial markers CK8 and CK18 [11, 12]. ILCs are usually ‘luminal’ type breast cancers that express the estrogen receptor (ER) gene and genes involved in ER activation, including the progesterone receptor (PR) gene [13, 14]. Cytosolic translocation of p120-catenin due to inactivation of E-cadherin is a hallmark of ILC, whereas classic and pleomorphic ILCs do not over-express the epidermal growth factor receptor (EGFR) gene [1, 15, 16]. While most classic ILCs lack expression of HER2 (*ERBB2*) [1], up to 81 % of pleomorphic ILCs express HER2 [17]. Moreover, although the somatic *TP53* mutation frequency in pleomorphic ILC may be as high as 46 %, this is a rare event in classic ILC (approximately 6 %), suggesting a role for p53 loss in the etiology of pleomorphic ILC [17–19]. These findings are supported by observations in mammary-specific E-cadherin and p53 knock-out mice that develop a mouse variant of pleomorphic ILC [6]. Furthermore, in contrast to classic ILC, pleomorphic ILC often expresses the apocrine differentiation marker gross cystic disease fluid protein 15 (GCDFP15) and the androgen receptor (AR) [17, 20]. The origin of pleomorphic ILC tumors is still under debate and, since results from conditional mouse models have suggested that all lobular cancer types are evolutionary linked (reviewed in [21]), It is currently unclear whether pleomorphic ILC is a dedifferentiated form of classic ILC or whether it evolves from ductal type tumors. The differential diagnosis between these breast cancer subtypes is important because surgery planning of ILC requires pre-operative MRI, due to an often more diffuse and multifocal growth pattern of lobular tumors and a higher incidence of contralateral tumors [22].

In cancer, DNA methylation is often disturbed and can act as a driving force during tumor progression [23, 24] (reviewed in [25]). DNA methylation occurs by the enzymatic transfer of a methyl group onto the carbon-5 position of a cytosine (often part of a cytosine phosphate guanosine (CpG) dinucleotide), which can result in gene silencing [26]. Promoter hypermethylation of tumor suppressor genes is considered to be an early event in carcinogenesis since high methylation levels have been found in columnar cell lesions, the earliest recognized breast cancer precursors [27]. Hence, methylation patterns may give insight in tumor progression and, thus, shed light on the precursor origin of pleomorphic ILC tumors. In light of the possible future extrapolation to methylation detection in biopsy, blood, nipple fluid and urine samples, DNA hypermethylation is a promising area in the clinical biomarker field. DNA hypermethylation analyses can be performed on formalin-fixed tissues and, thus, are suited for molecular profiling and the identification of markers that predict therapeutic responsiveness.

Here we have identified promoter methylation patterns in pleomorphic ILC in relation to ILC and IDC to identify pleomorphic ILC biomarkers. Methylation was assessed by methylation-specific multiplex ligation-dependent probe amplification (MS-MLPA), a highly reproducible technique that only requires small amounts (10 ng) of short DNA fragments and that shows high concordance with other established techniques such as quantitative multiplex methylation-specific PCR [28, 29]. MS-MLPA can be used in samples with mixed populations of cells. As long as 30 % of methylated DNA/tumor DNA is present in the sample, the methylation status will be recognized correctly [30]. We assessed the promoter methylation status of 24 tumor suppressor genes and compared 16 pleomorphic ILC, 20 classic ILC and 20 IDC cases. We found that the methylation patterns of classic ILC and IDC were comparable, and that the classic ILC and IDC profiles were mildly different from pleomorphic ILC. Furthermore, we found that the methylation status of the *RASSF1A*, *TP73*, *MLH1* and *BRCA1* gene promoters can be used as stratification markers to distinguish pleomorphic ILC from classic ILC and IDC.

## Materials and methods

### Patient material

Patient samples were derived from the archives of the Departments of Pathology at the University Medical Centre Utrecht, the Radboud University Medical Centre, Nijmegen, The Netherlands, the Institute of Pathology, Paderborn, Germany, and the Department of Pathology, Bács-Kiskun County Teaching Hospital, Kecskemét, Hungary. The clinicopathological characteristics of the patient samples are provided in Table 1. Classic and pleomorphic ILC and IDC cases were selected based on examination of haematoxylin and eosin (H&E)-stained slides by at least two pathologists. The use of left-over material was approved by the Tissue Science Committee of the UMC Utrecht [31]. Histological grades were assessed according to the Nottingham modification of the Scarff-Bloom-Richardson grading system [32]. ER and PR were considered positive when ≥10 % of the cells showed positive nuclear staining. HER2 was scored according to the modified DAKO scoring system, where only a score of 3+ was considered positive. The mitotic activity index (MAI) was assessed as reported before [33].

**Table 1:** Clinicopathological characteristics of breast cancer patients

		Classic ILC	Pleomorphic ILC	IDC
Feature	Grouping	N (%)	N (%)	
N		20	16	20
	Range	52–88	43–80	44–87
Histological grade	1	8 (40.0)	0 (0.0)	5 (25.0)
	2	6 (30.0)	5 (31.3)	5 (25.0)
	3	5 (25.0)	11 (68.8)	10 (50.0)
	Not available	1 (5.0)	0 (0.0)	–
MAI (%)	Mean	3	20	16.5
	Range	0–26	9–100	0–8
	Not available	1 (5.0)	1 (6.3)	–
Lymph node status	Negative^a^	11 (55.0)	8 (50.0)	7 (35.0)
	Positive^b^	7 (35.0)	8 (50.0)	13 (65.0)
	Not available	2 (10.0)	–	–
Receptor status	ER positive	19 (95.0)	14 (87.5)	15 (75.0)
	PR positive	10 (50.0)	10 (62.5)	13 (65.0)
	Her2 positive	0 (0.0)	0 (0.0)	3 (15.0)
Tumor size (cm)	≤2.0	1 (5.0)	4 (25.0)	8 (40.0)
	>2.0	18 (90.0)	12 (75.0)	12 (60.0)
	Not available	1 (5.0)	–	–

^a^: negative = N0 or N0(i+); ^b^: positive = ≥N1mi (according to TNM 7th edition, 2010)

### Methylation-specific multiplex ligation-dependent probe amplification

H&E stained sections were used to reveal pre-invasive lesions, necrosis and admixed lymphocytic infiltrates and to guide micro-dissections for DNA extraction. Areas with necrosis, dense lymphocytic infiltrates and pre-invasive lesions were intentionally avoided. All areas selected for micro-dissection had a tumor percentage of at least 70 %. Tumor tissue was derived from 4 μm thick sections (5 to 10, formalin-fixed paraffin embedded) and DNA was isolated by overnight incubation in lysis buffer (50 mM Tris–HCl, pH 8.0; 0.5 % Tween 20) with proteinase K (10 mg/ml, Roche) at 56 °C, followed by boiling for 10 min. After a 5 min centrifugation step (12,000 g), 5 μl supernatant was used for MLPA analysis according to the manufacturer’s instructions, using the ME001-C2 methylation kit (MRC-Holland). The principle of MS-MLPA has been described elsewhere [28] and the PCR and data analysis procedures were performed as reported before [27]. The ME001-C2 MS-MLPA probe mix contains 26 probes, detecting the methylation status of promoter CpG sites of 24 established and putative tumor suppressor genes (Table 2) that are frequently silenced by hypermethylation in tumors, but not in blood-derived DNA of healthy individuals. In addition, we included 15 reference probes. The cumulative methylation index (CMI) was calculated as the sum of the methylation percentage of all genes, as described before [34].

**Table 2:** Probes directed against the CpG islands of 24 tumor suppressor genes in the MS-MLPA kit (ME001-C2, MRC-Holland)

Length	Gene	Chromosome	Mapview	Full name
142	TIMP3	22q12.3	22-031.527795	TIMP metallopeptidase inhibitor 3
148	APC	5q22.2	05-112.101357	Adenomatosis Polyposis Coli
161	CDKN2A	9p21.3	09-021.985276	Cyclin-Dependent Kinase Inhibitor 2A
167	MLH1_a^a^	3p22.2	03-037.009621	MutL Homolog 1
184	ATM	11q22.3	11-107.599044	Ataxia Telangiectasia Mutated
193	RARB	3p24.2	03-025.444559	Retinoic Acid Receptor, beta
211	CDKN2B	9p21.3	09-021.998808	Cyclin-Dependent Kinase Inhibitor 2B
220	HIC1	17p13.3	17-001.905107	Hypermethylated In Cancer 1
238	CHFR	12q24.33	12-131.974372	Checkpoint with Forkhead and Ring finger domains
246	BRCA1	17q21.31	17-038.530811	Breast Cancer 1
265	CASP8	2q33.1	02-201.830871	Caspase 8
274	CDKN1B	12p13.1	12-012.761863	Cyclin-Dependent Kinase Inhibitor 1B
292	PTEN	10q23.3	10-089.612348	Phosphatase and Tensin homolog
301	BRCA2	13q12.3	13-031.787722	Breast Cancer 2
319	CD44	11p13	11-035.117389	CD44 molecule
328	RASSF1A_a^a^	3p21.31	03-050.353298	Ras Association (RalGDS/AF-6) domain Family member 1
346	DAPK1	9q21.33	09-089.303075	Death-Associated Protein Kinase 1
353	VHL	3p25.3	03-010.158426	Von Hippel-Lindau tumor suppressor
373	ESR1	6q25.1	06-152.170883	Estrogen Receptor 1
382	RASSF1A_b*	3p21.31	03-050.353347	Ras Association (RalGDS/AF-6) domain Family member 1
400	TP73	1p36.32	01-003.558977	Tumor Protein p73
409	FHIT	3p14.2	03-061.211918	Fragile Histidine Triad
427	CADM1	11q23.3	11-114.880585	Cell Adhesion Molecule 1
436	CDH13	16q23.3	16-081.218219	Cadherin 13
454	GSTP1	11q13.2	11-067.107774	Glutathione S-transferase pi 1
463	MLH1_b^a^	3p22.2	03-037.010000	MutL Homolog 1

^a^ For these genes two probe sets against different CpG sites (a and b) are present

### Statistics

Statistic calculations and ROC curve analyses were performed using IBM SPSS statistics v20.0 (SPSS Inc., Chicago, IL, USA). Two-sided *p* < 0.05 was considered significant. Absolute methylation levels were used to calculate *p*-values upon comparing classic ILC, pleomorphic ILC and IDC samples, using the Student’s *t*-test or Mann–Whitney *U* Test, and the Kruskal-Wallis test. Through Bonferroni-Holm correction of all *p*-values we excluded false-positives caused by multiple comparisons. Logistic regression analysis was used to reveal the best (combination of) genes able to discriminate pleomorphic ILC from classic ILC and/or IDC. A backward stepwise method was used until the most predictive variables remained. Unsupervised hierarchical clustering (Euclidean metric) using the statistical program R was performed on Z-scores to identify relevant clusters.

## Results and discussion

**Fig. 1:**
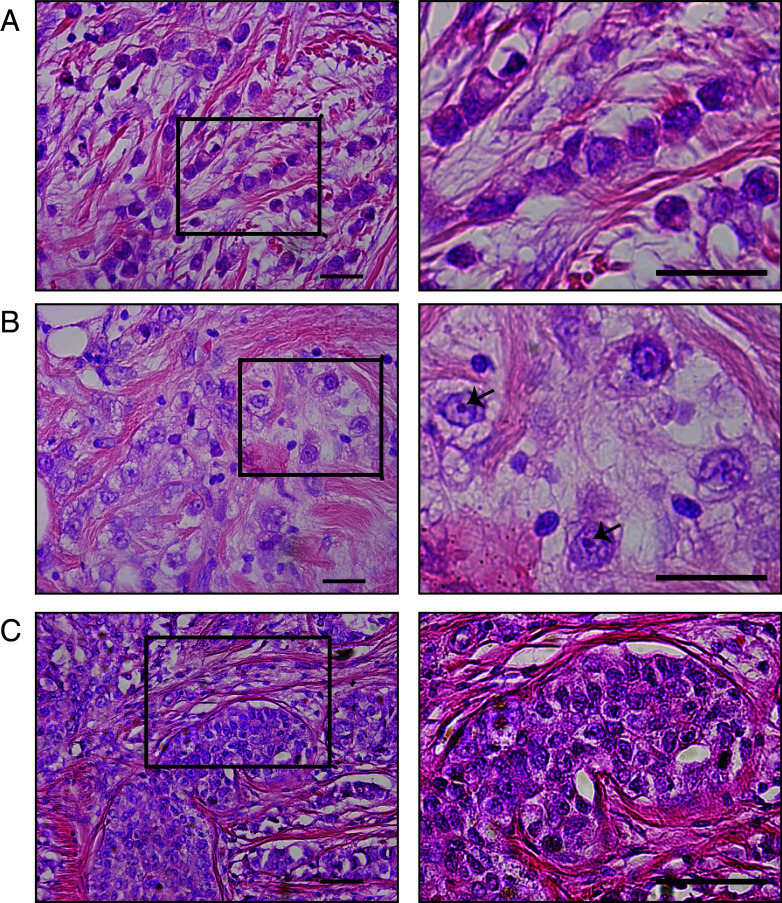
Representative H&E images of classic and pleomorphic ILC and IDC. Classic ILC is characterized by small regular cells, small nuclei and a low mitotic rate (**a**). The formation of single (‘indian’) files is a common characteristic of classic ILC (enlarged in right image). Pleomorphic ILCs display polygonal cells and frequent mitoses (**b**). The nuclei are often eccentric, highly pleomorphic and show distinctive nucleoli (enlarged in right image, arrows). IDC tumors are not characterized by specific features like ILC (**c**). In contrast to ILC, IDC often shows formation of ducts within the tumor (left and right image). All size bars indicate 25 μm

**Fig. 2:**
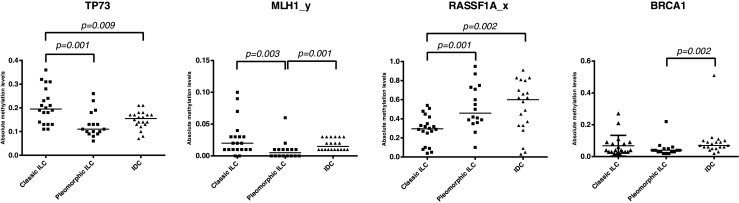
Methylation differences between classic and pleomorphic ILC. Scatter dot plots of the absolute methylation values that were found to be significantly different between the three breast cancer types. *TP73*, *MLH1*_*y and RASSF1A*_*x* were significantly different between pleomorphic and classic ILC, while only *MLH1*_*y* and *BRCA1* were significantly different between pleomorphic ILC and IDC. All *p*-values are derived from the Mann–Whitney analysis. The horizontal bars represent the median

Kruskal-Wallis one-way ANOVA analysis was carried out to assess differential methylation patterns in our non-parametric methylation data of the three breast cancer subtypes, i.e., classic ILC, pleomorphic ILC and IDC (Fig. 1; patient samples listed in Table 1). Sixteen of the 24 genes tested (listed in Table 2), including *BRCA1*, showed significant differences between the groups. However, after correction for multiple comparisons only the methylation patterns of *TP73* (*p* < 0.002), *MLH1*_*b* (*p* < 0.002) and *RASSF1A*_*x* (*p* < 0.002) were found to be significantly different between the three breast cancer subtypes (Fig. 2).

A (post hoc) Mann–Whitney test followed by multiple comparisons correction was carried out, using the 16 genes derived from the above Kruskal-Wallis analysis, to specify the differences between classic and pleomorphic ILCs. By doing so, we found that the methylation patterns of *TP73*, *MLH1*_*y* and *RASSF1A*_*x* were significantly different between the classic and pleomorphic ILCs. When compared to classic ILCs, pleomorphic ILCs showed less promoter methylation of the *MLH1*_*y* (*p* = 0.003) and *TP73* (*p* = 0.001) genes (Fig. 2), while the promoter methylation of the *RASSF1A* gene was found to be higher in pleomorphic ILCs (*p* = 0.001). The CMI of the pleomorphic ILCs was not significantly different from that of classic ILCs (353.3 versus 390.0, respectively; *p* = 0.437). In logistic regression analyses *TP73* (*p* = 0.017) and *RASSF1A* (*p* = 0.005) showed a joint independent discriminative value for pleomorphic ILCs versus classic ILCs (area under the curve (AUC) 0.888, CI 0.764-1.000, *p* < 0.001), with a combined receiver operating characteristic (ROC) curve-based sensitivity and specificity of 81 and 100 %, respectively.

After correction for multiple comparisons, we found that the methylation levels of both *MLH1*_*y* (*p* = 0.001) and *BRCA1* (*p* = 0.002) were significantly lower in pleomorphic ILCs than in IDCs (Fig. 2). The mean CMI of pleomorphic ILCs was not significantly different from that of IDCs (353.3 vs. 392.6, respectively; *p* = 0.357), indicating that the overall methylation patterns of these two breast cancer subgroups were similar. Logistic regression analysis showed that only *BRCA1* methylation (*p* = 0.002) had an independent discriminative value for pleomorphic ILC versus IDC (area under the curve (AUC) 781, CI 0.623-0.939, *p* = 0.004), with a ROC curve-based sensitivity and specificity of 75 and 81 %, respectively.

**Fig. 3:**
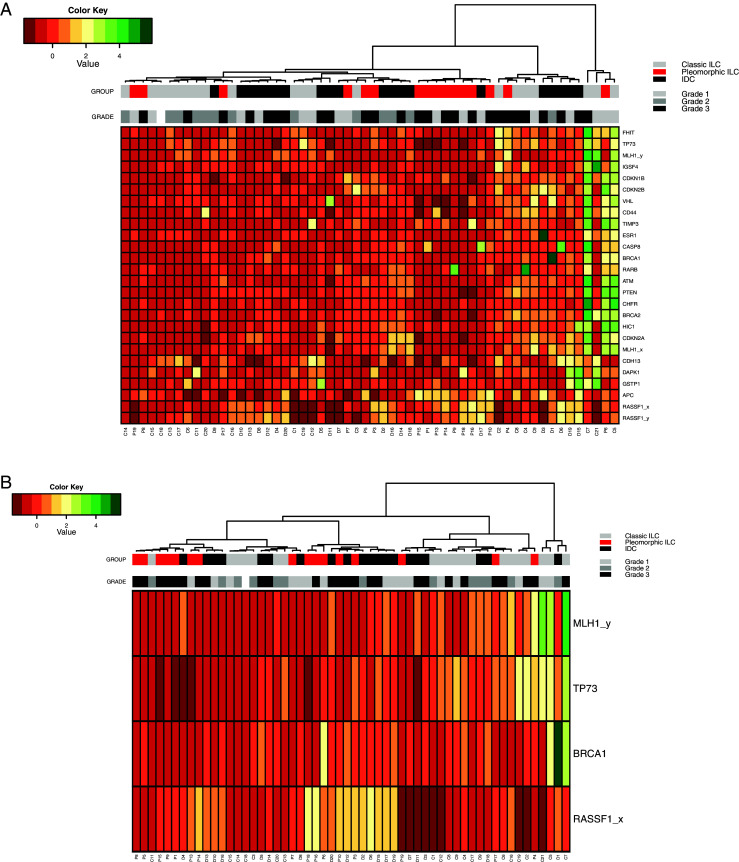
Cluster analysis of the breast cancer methylation data. (**a**) Hierarchical cluster analysis of Z-scores based on absolute methylation values by MS-MLPA of all interrogated genes in classic ILC (*light grey*), pleomorphic ILC (*red*) and IDC (*black*). (**b**) Hierarchical cluster analysis of Z-scores based on absolute methylation values of the four significantly differentially methylated genes according to the Mann–Whitney analysis

In order to determine if the absolute MS-MLPA values of our samples clearly defined our three breast cancer subtypes, we performed hierarchical Euclidean cluster analysis on all genes tested (Fig. 3a) and on the four genes that showed significant differences in the Mann–Whitney tests (Fig. 3b). Both cluster analyses revealed some clustering of pleomorphic ILC samples with IDC samples, while classic ILC samples usually formed separate clusters with other IDC samples.

The different advantages and possibilities of DNA methylation analyses for disease stratification and prognostication have led to a large amount of reports in the literature, also on breast cancer. However, the vast majority of these reports has so far focused on IDC, and we are unaware of any report on DNA methylation in pleomorphic ILC. Interestingly, in two reports the DNA methylation patterns were compared in ILC and IDC, and it was found that they were very similar in these two breast cancer subtypes [35, 36]. Our results are in agreement with this notion, while pleomorphic ILC appeared to exhibit a distinct methylation pattern with a CMI similar to that of classic ILC and IDC.

*BRCA1*, *MLH1* and *RASSF1A* are established tumor suppressor genes and *TP73* is a putative tumor suppressor gene. *BRCA1* and *MLH1* are involved in DNA repair and their functional loss causes an accumulation of gene defects. Interestingly, we found a significant association between the presence of *MLH1*_*x* (*p* = 0.013) and *BRCA1* (*p* = 0.013) promoter methylation and a high MAI (>12), and between *MLH1*_*x* and *BRCA1* promoter methylation (*p* = 0.004) (data not shown). Previously, *BRCA1* promoter methylation has been observed in 10–15 % of all sporadic breast cancer patients [37, 38]. Only 4–5 % of the lobular breast tumors carry a deleterious *BRCA1* mutation [39], and none of the 11 previously analyzed ILC samples showed *BRCA1* hypermethylation [37]. As we found that the *MLH1* and *BRCA1* methylation levels were lower in pleomorphic ILC compared to IDC, they may not be suitable as therapeutic targets, but they may be used as biomarkers.

To test the reproducibility of MS-MLPA, we have previously taken along 10 primary breast tumor samples in duplicate in at least 8 separate MS-MLPA runs (unpublished data). By doing so, we found that the *TP73*, *MLH1*_*y*, *RASSF1A*_*x* and *BRCA1* probes have an average standard deviation of 0.01, 0.01, 0.05 and 0.02 per sample, respectively. Based on these findings, we anticipate that the between-group differences observed for *TP73* and *RASSF1A*_*x* are reproducible and reliable. The differences between groups observed for *MLH1*_*y* and *BRCA1* are, however, less pronounced and may be the result of technical variability. We, therefore, recommend validating these findings by an independent highly sensitive and quantitative technique.

*TP73* is subject to alternative splicing, and the use of an alternative promoter results in different p73 isoforms that exhibit contrasting effects on tumor development [40]. Although *TP73* promoter methylation has been correlated with a poor survival of breast cancer patients [41], this methylation also impairs binding of the transcriptional repressor ZEB1, which may result in an increase in *TP73* expression [42]. Unfortunately, studies reporting *TP73* methylation levels in normal breast tissue are scarce and not combined with protein or RNA expression analyses [27, 43], and *TP73* methylation studies in ILC have not been reported yet. As we found *TP73* promoter methylation to be relatively low in pleomorphic ILC compared to classic ILC, it may serve as a biomarker, whereas it is considered less suited as a target for therapy. Further studies are needed to determine the effect of *TP73* methylation on protein expression and to determine the functional consequences in pleomorphic ILC.

*RASSF1A* promoter methylation was found to be higher in pleomorphic ILC than in classic ILC. Although uncommon, *RASSF1A* polymorphisms and deletions have been encountered and *RASSF1A* promoter hypermethylation has been found to frequently occur in different tumor types [44]. About 70–85 % of ILC and IDC cases show *RASSF1A* hypermethylation [35, 45]. Also, hypermethylation of *RASSF1A* in pre-operative serum of breast cancer patients has been found to serve as an independent prognostic marker correlated with a poor overall survival [46]. Since *RASSF1A* hypermethylation is rarely observed in normal breast tissues, it is considered to be an early event in breast cancer development [46, 47] and, as such, it may serve as a promising breast cancer biomarker. *RASSF1* is a member of the *RASSF* family of genes (*RASSF 1*–*8*), and gives rise to 8 different isoforms due to alternative splicing and alternative promoter usage [48]. Next to the RASSF proteins, RAF and phosphatidylinositol 3-kinase (PI3K) are also known as RAS effectors, i.e., proteins that specifically bind the GTP-bound form of RAS. In contrast to RAF and PI3K, which control proliferation and survival, the *RASSF* genes are known to act as tumor suppressors [48]. *RASSF1A* null-mice show an increased incidence of spontaneous tumor formation, a decreased survival rate and an increased susceptibility for mutagens (reviewed in [48]). In addition, it has been found that exogenous expressions of *RASSF1A* in different tumor cell lines reduces their viability, proliferation and invasion [48]. These findings, combined with our data showing increased *RASSF1A* promoter methylation in pleomorphic ILC, renders *RASSF1A* into an interesting and functional biomarker for lobular breast cancer.

In conclusion, our data indicate that the promoter methylation signature of the *TP73*, *MLH1*, *RASSF1A* and *BRCA1* genes may serve as a biomarker to distinguish pleomorphic ILC from classic ILC and IDC. Since pleomorphic ILC is considered to be an aggressive breast cancer variant, and since pre-operative MRI is favorable for ILC patients but not for IDC patients, pleomorphic ILC biomarkers may be useful for treatment design in cases where a pathological distinction between ILC and IDC is questionable. Future research is needed to confirm our findings in an independent patient group and to evaluate the potential of the respective methylation markers as therapeutic targets.
